# “*statcheck*”: Automatically detect statistical reporting inconsistencies to increase reproducibility of meta‐analyses

**DOI:** 10.1002/jrsm.1408

**Published:** 2020-04-27

**Authors:** Michèle B. Nuijten, Joshua R. Polanin

**Affiliations:** ^1^ The Department of Methodology and Statistics, Tilburg University Tilburg The Netherlands; ^2^ Research & Evaluation, American Institutes for Research Washington DC USA

**Keywords:** meta‐analysis, reporting standards, reproducibility, statcheck, statistical error

## Abstract

We present the R package and web app *statcheck* to automatically detect statistical reporting inconsistencies in primary studies and meta‐analyses. Previous research has shown a high prevalence of reported *p*‐values that are inconsistent ‐ meaning a re‐calculated *p‐*value, based on the reported test statistic and degrees of freedom, does not match the author‐reported *p*‐value. Such inconsistencies affect the reproducibility and evidential value of published findings. The tool *statcheck* can help researchers to identify statistical inconsistencies so that they may correct them. In this paper, we provide an overview of the prevalence and consequences of statistical reporting inconsistencies. We also discuss the tool *statcheck* in more detail and give an example of how it can be used in a meta‐analysis. We end with some recommendations concerning the use of *statcheck* in meta‐analyses and make a case for better reporting standards of statistical results.

**Highlights**

- Reporting inconsistencies where the reported *p*‐value does not match the degrees of freedom and test statistic are widespread.
- The R package and web app *statcheck* can automatically detect statistical reporting inconsistencies in meta‐analyses.
- If meta‐analysts adhere to APA reporting style, *statcheck* provides a quick and easy tool to detect reporting inconsistencies and increase reproducibility.

## 1 INTRODUCTION

Researchers in the health and social sciences continue to draw conclusions in the health and social sciences based solely on Null Hypothesis Significance Tests (NHST).^1^, ^2^, ^3^, ^4^ Primary study authors use these tests often, yet meta‐analysts use them as well: NHST results in primary studies can also be used to calculate effect sizes to include in meta‐analyses, and a recent review of meta‐analyses published in the social sciences^5^ revealed that the average review conducted nearly 60 NHSTs. NHSTs can therefore lead to policy and practice decisions, and as such, their accuracy is paramount.

Extant evidence suggests that statistical reporting errors are widespread. A recent review of significance testing in primary studies found that one in eight primary studies published in eight high‐profile psychology journals had “grossly inconsistent *p*‐values that may have affected the statistical conclusion”.^6^ The authors applied the phrase “grossly inconsistent” to represent cases in which conclusions of the significance test would change based on a recalculation of the *p*‐value. For example, a study's author said a *p*‐value was <.05 but the test statistic and degrees of freedom indicated the *p*‐value was actually >.05, or vice versa. An alarmingly high number of impactful results of statistical significance tests were inconsistent and potentially misleadingly inaccurate, too: the results indicated that gross inconsistencies favored statistically significant results.

Detecting statistical reporting inconsistencies is time‐consuming and, ironically, error‐prone work. Because of that, Epskamp and Nuijten^7^ developed the R package *statcheck*: an automated tool to extract NHST results from articles and recalculate *p*‐values. Recently, Polanin and Nuijten^8^ extended *statcheck*'*s* functionality to include tests often used in meta‐analyses. In this paper, we elaborate on how *statcheck* can be useful in the context of meta‐analysis. We give a brief overview of the prevalence and consequences of statistical reporting inconsistencies based on a review of 402 meta‐analyses. We also discuss the tool *statcheck* in more detail and give an example of how it can be used in a meta‐analysis. We end with some recommendations concerning the use of *statcheck* in meta‐analyses and make a case for better reporting standards for statistical results.

## 2 WHY SHOULD RESEARCH SYNTHESISTS CARE ABOUT STATISTICAL REPORTING INCONSISTENCIES?

We focus on a specific type of statistical error: statistical reporting inconsistencies, where the reported *p*‐value does not match the accompanying test statistic and degrees of freedom. Statistical reporting inconsistencies are harmful for several reasons. First, these inconsistencies can lead to wrong substantive conclusions when the reported *p*‐value is significant whereas the recalculated *p*‐value is not, or vice versa. Second, statistical reporting inconsistencies can also be symptoms of deeper, underlying problems. Reporting inconsistencies, for example, could signal human error, sloppiness,^9^ or questionable research practices.^10^ Third, regardless of their cause, statistical inconsistencies affect the overall reproducibility of a paper: the ability to obtain the same numbers with the same data and analyses. Results that appear erroneous and that cannot be reproduced by reanalysis are unreliable and, worse, might be considered invalid.^11^

Statistical reporting inconsistencies can also affect the quality of meta‐analyses in various ways. From the perspective of the primary studies included, reported NHST results can be used to calculate effect sizes to include in a meta‐analysis: reported results of *t* tests or *F* tests can be converted to Cohen's *d*. However, if the results of these NHSTs are inconsistent, it is possible that the test statistics are incorrect (e.g., a typo in a *t*‐value). If that erroneous test statistic is then used to calculate the effect size to include in the meta‐analysis, the eventual meta‐analytic effect size will also contain error.^12^ Furthermore, from the perspective of the meta‐analytic results, the reported NHSTs of meta‐analytical averages, heterogeneity tests, and moderator analyses remain widely reported and widely used when drawing conclusions. As a result, the results of these statistical tests require additional scrutiny.

## 3 INTRODUCING “statcheck” AS A SOLUTION FOR META‐ANALYSES

To detect statistical reporting inconsistencies, Epskamp and Nuijten^7^ developed the R package *statcheck*, with an accompanying web app at https://statcheck.io.^13^ statcheckis a free and easy‐to‐use tool that automatically extracts statistical results from articles and recomputes *p*‐values to check their internal consistency. *statcheck* was developed to check results in primary studies, and we recently extended its functionality to meta‐analyses.^8^

### 3.1 How does statcheck work?

The algorithm behind *statcheck* consists of four steps. First, *statcheck* converts an article (or a folder of articles) from PDF or HTML to plain text. Second, using regular expressions, *statcheck* searches for specific combinations of letters, numbers, and symbols that signal the presence of an NHST result. Polanin and Nuijten^8^ updated *statcheck* to recognize *Q* tests in addition to the original recognition of *t*, *F*, *χ* ^*2*^, *Z*, and correlations that are reported in the full text according to APA style (e.g., *t*(28) = 2.14, *p* = .04; 14). Third, *statcheck* uses the reported test statistic and degrees of freedom to recalculate the *p*‐value. Fourth, it compares the reported and computed *p*‐value to see if they match. If they do not match, the result is flagged as an “inconsistency.” If the reported *p*‐value is significant and the computed *p*‐value is not, or vice versa, the result is flagged as a “gross inconsistency.” By default, *statcheck* assumes an *α* of .05, but this can be manually adjusted.

In flagging inconsistencies (or gross inconsistencies), *statcheck* takes rounding into account. A test statistic reported as *t* = 2.5, for example, could correspond to actual *t*‐values ranging from 2.45 to 2.54. *statcheck* will consider all *p*‐values as consistent if they belong to that range of possible test statistics. *statcheck* can also take one‐tailed testing into account. If *statcheck* finds the word one‐tailed, one‐sided, or directional in the full text, *and* the reported *p*‐value would have been correct if it belonged to a one‐tailed test, *statcheck* flags the result as consistent.

### 3.2 *statcheck*'*s* accuracy and limitations

*statcheck* is specifically designed to recognize and check statistics reported in APA style in full text. This means that *statcheck* will not recognize statistics reported with deviations from APA style. Furthermore, *statcheck* will often not recognize statistics reported in tables, because statistics in tables are often not fully reported (e.g., the degrees of freedom for the entire table are in the table caption, rather than next to each test statistics and *p*‐value).

*statcheck* can detect statistics in both PDF and HTML files. However, the conversion of PDF to plain text is less reliable than HTML to plain text. This has to do with the wide variety of typesetting and text encoding in different journals. We therefore recommend to use HTML files, where possible.

In flagging (gross) inconsistencies, *statcheck*'s accuracy is high. In a previous study,^14^ *statcheck*'s performance was compared with manual coding, and it was concluded that *statcheck*'s sensitivity (true positive rate) and specificity (true negative rate) were high: between 85.3% and 100%, and between 96.0% and 100%, respectively, depending on the assumptions and settings. The overall accuracy of *statcheck* ranged from 96.2% to 99.9%. (for details, see Ref.^14^)

It is important to note that statistical inconsistencies can arise when some (but not all) of the elements of a reported results are adjusted for multiple testing, post hoc testing, or possible violations of assumptions. For example, to correct for multiple testing, authors often multiply the *p*‐value by the number of tests performed (a procedure tantamount to a Bonferroni correction). However, such a multiplied *p*‐value is then no longer consistent with the original, uncorrected, test statistic, and degrees of freedom. Similar inconsistencies can arise when authors adjust for violations of the sphericity assumption by reporting corrected degrees of freedom in combination with the uncorrected test statistic and *p*‐value. *statcheck* will flag such cases as inconsistencies. To avoid inconsistencies due to statistical corrections, we recommend that authors report the fully adjusted result (ie, the corrected degrees of freedom and the accompanying corrected test statistic and *p*‐value), or, in the case of a Bonferroni correction, to divide their α by the number of tests performed, instead of multiplying the *p*‐value.

### 3.3 Using *statcheck* in meta‐analyses

NHST results are ubiquitous in meta‐analyses.^5^ It is imaginable that the high prevalence of statistical reporting inconsistencies in primary studies also translates to meta‐analyses. To test this empirically, we adapted *statcheck* to also pick up NHST results in meta‐analyses.^8^

The types of statistical significance test that occur most in meta‐analyses are tests of the overall effect size, tests of homogeneity and heterogeneity, subgroup analyses, and meta‐regressions. In most cases, the test statistics belonging to these analyses are *Z*, *χ* ^*2*^, *t*, and *F*, which *statcheck* could theoretically already extract. One exception is the *Q* test for heterogeneity. Even though the *Q* test follows a *χ* ^*2*^‐distribution, previous versions of *statcheck* would not recognize it if it is reported with the statistic *Q*. To solve this, we adapted *statcheck* to recognize *Q* tests as well. *statcheck* recognizes the following types of *Q* tests: identifying heterogeneity (*Q* omnibus), and explaining heterogeneity (*Q*_*within*_ or *Q*_*w*_, and *Q*_*between*_ or *Q*_*b*_).

After updating *statcheck*, we used it to analyze 402 meta‐analyses published in the social sciences. Our sample derived from three locations used in previous meta‐reviews^1^: Campbell Collaboration reviews published on or before May 2017 (*n* = 135) and used in Polanin and Nuijten^2^, ^8^; reviews published in the *Review of Educational Research* or *Psychological Bulletin* on or before May 2013 and used in Polanin and Pigott^5^ (*n* = 137)^5^; and^3^ reviews on intelligence and IQ, found by searching the ISI Web of Knowledge and published on or before August 2014, used in Nuijten and colleagues (2018)^15^ (*n* = 130). The results of using *statcheck* on this sample revealed that, of the 87 meta‐analyses with NHST results reported in APA style in the full text, 39.1% contained at least one statistical inconsistency and 8% contained at least one gross inconsistency where the statistical conclusion may have changed. Previous analyses conducted on primary studies^6^ found a greater prevalence of inconsistences (50%) and gross inconsistencies (13%); however, the prevalence of inconsistences and gross inconsistencies in our sample remains concerning. The prevalence of APA‐reported statistics is also lower and potentially problematic, because it seemed to signal a lack of any formalized or consistent reporting style. See Polanin and Nuijten^8^ for a full explanation of the methods and results.

### 3.4 How to use *statcheck* in R or in a browser

*statcheck* can be used as an R package^7^ or as a web app at https://statcheck.io.^13^ To use the *statcheck* R package, you first need to download a program called Xpdf, which converts PDF files into plain text. Xpdf is free and can be downloaded from http://www.xpdfreader.com/download.html. The binaries of this program need to be added to the system path. For detailed instructions on how to do this, see the *statcheck* manual at https://rpubs.com/michelenuijten/statcheckmanual.

After Xpdf is installed, *statcheck* can be installed from CRAN and loaded in R as follows:


install.packages(“statcheck”)

library(statcheck)





*statcheck* can be used on a string of text, on a PDF or HTML file, or on an entire folder of PDF and/or HTML files as follows:


# check a string of text

statcheck(“Qb(1) = 3.78, p < .05”)



# check a PDF or HTML article

checkPDF(“C:/MyDocuments/Research/Paper1.pdf”)

checkHTML(“C:/MyDocuments/Research/Paper1.html”)



# check all PDF and HTML articles in a directory

checkdir(“C:/MyDocuments/Research”)





**FIGURE 1:**
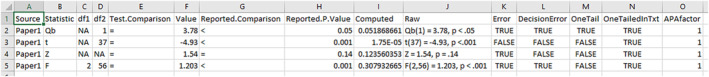
Example of the *statcheck* output for an article called “Paper1” [Colour figure can be viewed at wileyonlinelibrary.com]

All the functions above will print the same type of output to the console: a data frame where each row represents an extracted statistic. The data frame contains the extracted statistics, the recomputed *p*‐value, whether it is a (gross) inconsistency or not, and some additional variables. Figure 1 shows an example of the *statcheck* output for an article called “Paper1,” in which *statcheck* detected four hypothesis tests. In addition to the base analyses, the user can specify several options. It is possible, for example, to be more or less stringent with what *statcheck* will count as an inconsistency by accounting for one‐tailed testing, or to assume a different alpha‐level. The output includes the main variables of interest are the extracted statistic (“Raw” in the output), the computed *p*‐value (“Computed” in the output), and whether it is an inconsistency (“Error” in the output), or gross inconsistency (“DecisionError” in the output). Note that when “Error = TRUE,” this means that the result is inconsistent.

**FIGURE 2:**
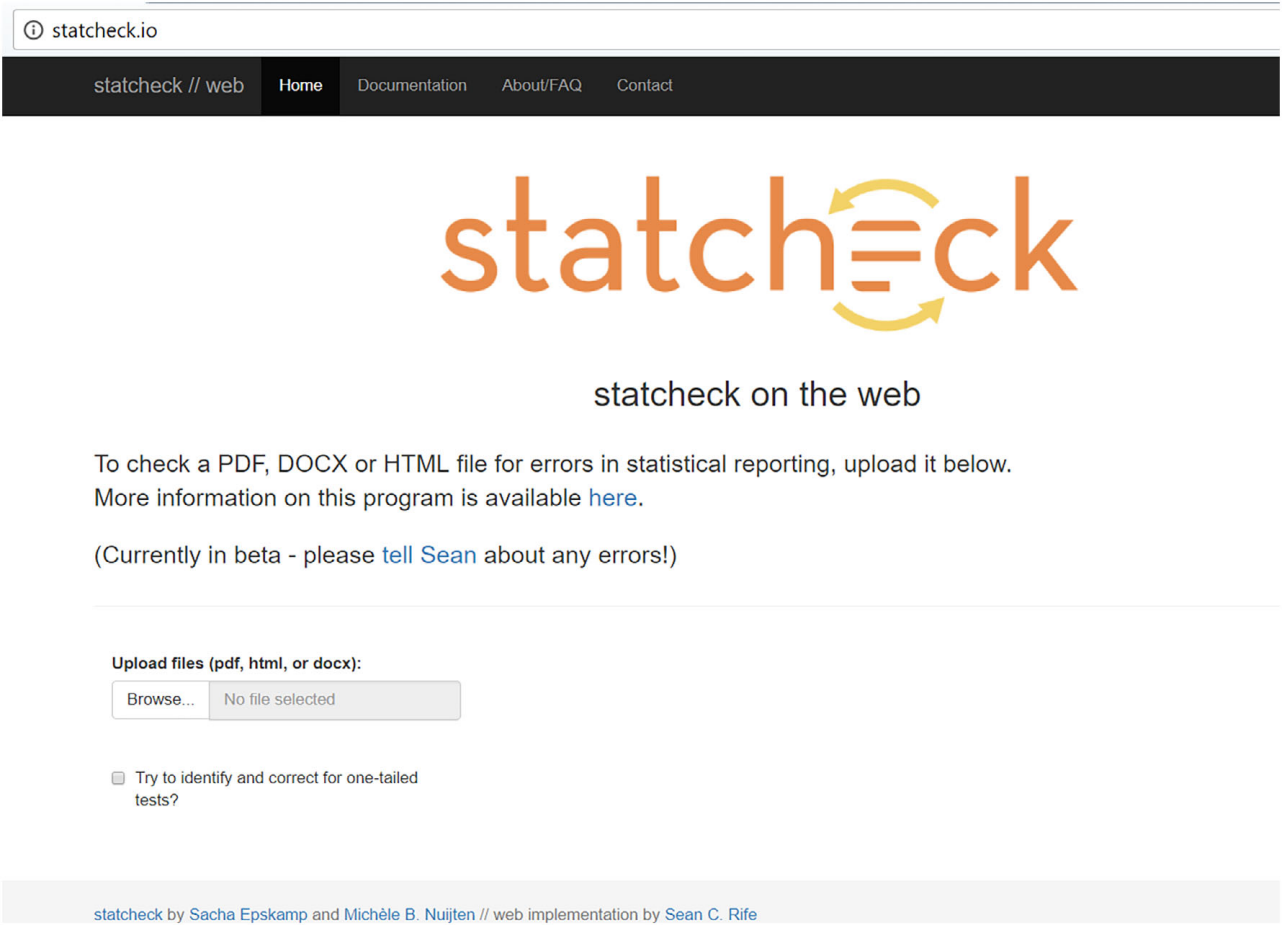
Screenshot of the *statcheck* web app at http://statcheck.io [Colour figure can be viewed at wileyonlinelibrary.com]

Alternatively, a meta‐analysts could also use *statcheck* in a browser via http://statcheck.io.^13^ This user‐friendly app requires no programming skills and merely asks the user to upload a paper to check for inconsistencies (see Figure 2). The app also accepts papers in .docx format in addition to PDF and HTML files, but cannot be used to check an entire directory at once.

**FIGURE 3:**
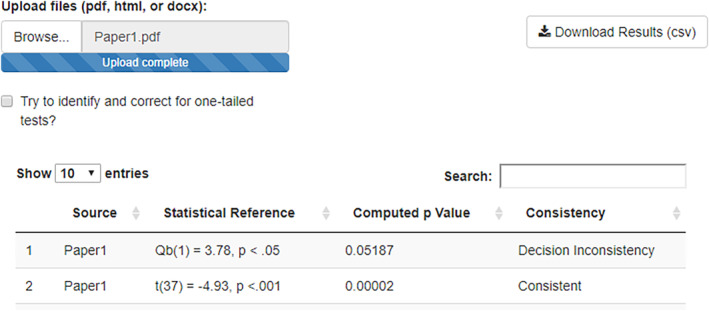
Screenshot of the output of the *statcheck* web app [Colour figure can be viewed at wileyonlinelibrary.com]

Once the meta‐analyst uploads a paper via “Browse,” a more concise version of the output, compared to the R package, is displayed (see Figure 3). The more extensive version of the output can be downloaded in CSV format with the button in the top right corner. The output in the browser identifies the source, the statistical test, the *statcheck* computed *p*‐value, and whether the computed *p*‐value matches the reported *p*‐value. For more information on both the browser and R package versions of *statcheck*, please see the *statcheck* manual at https://rpubs.com/michelenuijten/statcheckmanual/

### 3.5 Plans for further development

We routinely update *statcheck* to improve its performance and increase functionality. Some concrete plans for future updates include a feature on the web app to allow users to simply copy‐paste a statistical result they want to check, and the option to also check .docx files with the R package. Furthermore, a new PDF to text converter is being tested, so that users do not have to download and install the program Xpdf anymore when they want to install *statcheck*. The latest development can be followed on GitHub at https://github.com/MicheleNuijten/statcheck.

## 4 RECOMMENDATIONS

We make two broad recommendations for meta‐analytic practice. The first is simply that meta‐analysts should strive to report statistical results completely and systematically, preferably using widely‐adopted reporting guidelines such as the APA guidelines.^16^ If researchers always report statistics in the same way, it is easier for readers to quickly filter out important information and quicker for meta‐analysts attempting to locate vital information. The second recommendation is to use *statcheck* as a way to double check the reporting of results. While we recognize that recommending our product serves to further the use of the product and our research, we believe that *statcheck*, and perhaps additional programs like it, can help decrease the number of statistical reporting errors and increase the reliability of results. Editors of journals that focus on meta‐analyses could also consider making *statcheck* a standard part of their peer review process (following the journals *Psychological Science* and the *Journal of Experimental Social Psychology*).

Meta‐analysts can use *statcheck* to detect potential inconsistencies in their meta‐analysis, but also to detect inconsistencies in the primary studies they intend to include. Detecting inconsistencies in primary studies is especially relevant if the meta‐analyst needs to calculate the effect size based on reported NHST results. However, even if the effect size could be literally copied from the primary paper, it could be useful to scan a paper for statistical inconsistencies. If *statcheck* flags many NHST results as inconsistent, it could reflect something about the overall statistical quality of the paper. Meta‐analysts might consider recalculating the effect size from the raw data, to avoid any errors in the included effect size.

## CONFLICT OF INTEREST

The authors reported no conflict of interest.

## Data Availability

Data sharing is not applicable to this article as no new data were created or analyzed in this study.
